# Recommendations for determining HPV status in patients with oropharyngeal cancers under TNM8 guidelines: a two-tier approach

**DOI:** 10.1038/s41416-019-0414-9

**Published:** 2019-03-20

**Authors:** Stephanie G. Craig, Lesley A. Anderson, Andrew G. Schache, Michael Moran, Laura Graham, Keith Currie, Keith Rooney, Max Robinson, Navdeep S. Upile, Rachel Brooker, Mina Mesri, Victoria Bingham, Stephen McQuaid, Terry Jones, Dennis J. McCance, Manuel Salto-Tellez, Simon S. McDade, Jacqueline A. James

**Affiliations:** 1 Centre for Cell Research and Cell Biology, Queen’s University Belfast, Belfast, Northern Ireland UK; 2 Northern Ireland Cancer Registry, Belfast, Northern Ireland UK; 3 Centre for Public Health, Queen’s University Belfast, Belfast, Northern Ireland UK; 4 Department of Molecular and Clinical Cancer Medicine, University of Liverpool, Liverpool, England UK; 5 Pfizer Oncology, Walton Oaks, England UK; 6 Belfast Health and Social Care Trust, Belfast, Northern Ireland UK; 7 Centre for Oral Health Research, Newcastle University, England, UK; 8 Aintree University Hospital NHS Foundation Trust, Liverpool, England UK; 9 Department of Pathology, University of New Mexico Medical School, Albuquerque, NM 87131 USA

**Keywords:** Prognostic markers, Head and neck cancer

## Abstract

**Background:**

TNM8 staging for oropharyngeal squamous cell carcinomas (OPSCC) surrogates p16 immunohistochemistry for HPV testing. Patients with p16+ OPSCC may lack HPV aetiology. Here, we evaluate the suitability of TNM8 staging for guiding prognosis in such patients.

**Methods:**

HPV status was ascertained using p16 immunohistochemistry and high-risk HPV RNA and DNA in situ hybridisation. Survival by stage in a cohort of OPSCC patients was evaluated using TNM7/TNM8 staging. Survival of p16+/HPV− patients was compared to p16 status.

**Results:**

TNM8 staging was found to improve on TNM7 (log rank *p* = 0·0190 for TNM8 compared with *p* = 0·0530 for TNM7) in p16+ patients. Patients who tested p16+ but were HPV− (*n* = 20) had significantly reduced five-year survival (33%) compared to p16+ patients (77%) but not p16− patients (35%). Cancer stage was reduced in 95% of p16+/HPV− patients despite having a mortality rate twice (HR 2.66 [95% CI: 1.37–5.15]) that of p16+/HPV+ patients under new TNM8 staging criteria.

**Conclusion:**

Given the significantly poorer survival of p16+/HPV− OPSCCs, these data provide compelling evidence for use of an HPV-specific test for staging classification. This has particular relevance in light of potential treatment de-escalation that could expose these patients to inappropriately reduced treatment intensity as treatment algorithms evolve.

## Background

Transcriptionally active human papillomavirus (HPV) is the causal agent in 13–60% of oropharyngeal squamous cell carcinoma (OPSCC), with increasing incidence rates globally.^1,2^ HPV-related (HPV+) OPSCC exhibits significantly better prognosis than HPV negative (HPV−) OPSCC, and the two tumour classifications represent distinct molecular and clinical entities.^3^ Molecular stratification based on HPV status has recently been proposed to improve clinical management. The American Joint Committee on Cancer and the Union for International Cancer Control eighth edition TNM staging guidelines (TNM8), effective January 2018, recommend stratification of OPSCC by HPV status to improve staging.^4,5^ Due to insufficient evidence supporting the clinical need for a second line HPV test and for ease of clinical adoption, use of p16 immunohistochemistry (IHC) alone as a surrogate marker of HPV status has been recommended.^6,7^ p16 IHC is a highly sensitive test surrogate for transcriptionally active HPV+ OPSCC; however, recent analyses have identified that as many as 20% of p16+ OPSCC patients may lack transcriptionally active HPV with potential relevance to clinical outcomes.^8–11^ A recent meta-analysis for head and neck cancers determined incidence was approximately 6.7%.^3^ Hence, the United Kingdom (UK) Royal College of Pathologists recommend use of a second line in situ hybridisation test (ISH) for confirmation of HPV status in p16 positive (p16+) tumours.^12^

Despite early indications of improved outcomes, current clinical practice still dictates that all OPSCC patients are managed based on stage and other clinical parameters.^13^ At present, patients with OPSCC are only stratified by HPV status if they are enrolled in a clinical trial evaluating de-intensification of conventional treatment modalities or new agents.^13^ The outcomes of ongoing clinical trials for de-escalation of treatment based on p16 and/or an ISH test (to confirm HPV status) will make accurate classification and staging of patients increasingly important to ensure appropriate clinical management and prognostic predictions.^11^ The new TNM8 staging guidelines, reduces stage allocation of HPV+ tumours by using p16 IHC alone and has set the precedent for stratification of OPSCC. This has been reflected in the most recent recommendations on HPV testing guidelines published by the College of American Pathologists.^6^

The aim of this study was to assess the prognostic relevance of p16 positivity with and without transcriptionally active HPV in a large, non-biased, combined UK cohort of OPSCC patients. Three independent cohorts with similar baseline characteristics were combined to evaluate the staging capacity of TNM8 for p16+ patients and to assess the impact of TNM8 staging in p16+/HPV− patients.

## Materials and methods

### Patients

All consecutive OPSCCs diagnosed in Northern Ireland from 2000 to 2011 were included under ethical approval from the Northern Ireland Biobank (NIB 11/0001). Two distinct OPSCC cohorts diagnosed within the Liverpool Head and Neck Oncology Service from 1998 to 2009 and 2002 to 2011, respectively, were accessed under prior ethical approval (South Sefton Research Ethic Committee, EC·47·01–6; North West Five Research Ethics Committee, EC·09·H1010·5, REC 11/NQ/0452 and REC 16/LO/1726). Clinical data were retrieved from individual patient electronic clinical records. Data recorded for each cohort included sex, age, tumour stage, nodal status, distant metastases, treatment received, smoking status, alcohol history, date of diagnosis and death. Overall survival was defined as the time from diagnosis until time of death as recorded on the death certificate. Data were right censored for patients still alive using the following censor dates (Northern Ireland cohort: 05/12/2016, Liverpool cohort one: 24/11/2017, cohort two: 26/12/2017). Clinicians were unaware of HPV status at the time of treatment for patients in the cohort. Treatment decisions were made in accordance with contemporaneous UK national guidelines.

Formalin fixed paraffin embedded (FFPE) specimens (biopsies or surgical resections) arising from the oropharynx were identified from three independent UK OPSCC cohorts and used to construct tissue microarrays (TMAs) wherever possible. Tumour site of origin was determined in patients identified by clinical, pathological and radiological review prior to inclusion in the study. Inclusion criteria were based on confirmed diagnosis of an untreated primary OPSCC; here defined as either base of tongue (C01), soft palate (C05·1), uvula (C05·2) tonsils (C09), or oropharynx not otherwise specified (C10·9).

### Procedures

TMA sections were assessed for p16 IHC as previously described using a Ventana Benchmark Autostainer with a commercial kit (CINtec® p16 Histology); all assays were validated on full face sections.^2,14^ For each patient, triplicate cores were independently assessed by two investigators for all assays. Patients were considered p16 positive by p16 IHC if there was strong and diffuse nuclear and cytoplasmic staining in at least 70% of tumour cells.^15^

Positive (either *PPIB* or *UBC*) and negative (*DapB*) reagent controls were employed across TMAs and assessed prior to HPV RNA-ISH detection in order to determine integrity of mRNA within the TMA.^16^ Patient FFPE material positive by *PPIB* or *UBC* but negative for *DapB* were considered suitable for analysis by the test probe for HPV. For the Northern Ireland cohort, high-risk HPV RNA-ISH was carried out in the Northern Ireland Molecular Pathology Laboratory using RNAscope 2·0 manual assay with a pooled HPV genotype detection kit (Probe-HPV-HR18 recognising HPV 16, 18, 26, 31, 33, 35, 39, 45, 51, 52, 53, 56, 58, 59, 66, 68, 73, and 82 E6/E7 mRNA, Advanced Cell Diagnostics). RNA-ISH for the Liverpool cohort was carried out in the Newcastle upon Tyne Hospitals National Health Service Foundation Trust using the RNAscope 2·0 manual assay with a pooled HPV genotype detection kit (pooled HPV subtypes: Probe-HPV-HR7 recognising HPV 16, 18, 31, 33, 35, 52, and 58 E6/E7 mRNA, Advanced Cell Diagnostics). For both cohorts, patients were considered HPV RNA-ISH positive if the tumour tissue demonstrated brown reaction product specifically localising within the malignant nuclei.

Owing to differences in HPV genotypes represented in the RNA-ISH probes used in Northern Ireland and Liverpool, high-risk DNA-ISH was utilised in order to prevent bias in patients who presented with p16+/HPV− tumours. Patients were assessed for high-risk HPV DNA-ISH as previously described using a Ventana Benchmark Autostainer with a commercial kit (Inform HPV III family 16 probe (B)-(recognising HPV 16, 18, 31, 33, 35, 39, 45, 51, 52, 56, 58, and 66 subtypes).^2,14^ Patients were considered positive for HPV DNA-ISH if tumour tissue demonstrated blue reaction product specifically localising within the malignant nuclei.

### Statistical analysis

Patients were initially staged according to TNM7 guidelines with subsequent clinical restaging to TNM8 using p16 status. Cohort baseline characteristics were compared between p16+ and p16− OPSCC patients using Pearson’s chi-square test for independence.

Overall five-year survival analysis using the Kaplan–Meier method was conducted. Survival curves were compared using the log-rank test. The missing indicator method was used to handle missing clinical data. Cox proportional-hazards models were used to calculate hazard ratios (HR) and associated 95% confidence intervals (CI). Multivariable models were developed by backwards selection of age, sex, smoking status, alcohol history and treatment received.

The reporting standards of the current study fulfil recommendations set by the STROBE statement for reporting of observational studies and the REMARK guidelines for tumour biomarker prognostic studies.^17–19^

## Results

### Clinical cohort

The three cohorts included 521 primary OPSCCs in previously untreated patients for which HPV status was determined by p16 IHC and HPV RNA-ISH in 482 (93%) individuals. Of these, 446 (86%) patients had complete treatment and staging information for TNM7 and TNM8 and were therefore included in subsequent analyses. The proportion of p16+/HPV+ patients in the Liverpool cohorts was significantly higher than observed in the Northern Irish epidemiological cohort (47 and 66% vs. 37% in the Liverpool A, Liverpool B and Northern Ireland cohorts, respectively), Table 1. Interestingly, incidence of p16+/HPV− patients relative to p16 positivity within the Liverpool cohorts was lower than expected when compared to the population based Northern Ireland cohort (13 and 6% vs. 11% of p16+ cases were HPV− in the Liverpool A, Liverpool B and Northern Ireland cohorts, respectively). In spite of this, the three cohorts displayed similar baseline characteristics for age, sex, TNM7 and TNM8 staging, supporting combination of the cohorts for further analysis. As significant differences were identified between cohorts for smoking status, alcohol history, treatment received and tumour site of origin (*p* < 0.0009) in addition to p16/HPV status these were included in the multivariable survival analyses. Mean follow-up for the combined cohort was 5.04 years (Range 0.01–25.06 years).

**Table 1:** Baseline characteristics of study patients, according to cohort

	Study cohorts				
	Northern Ireland	Liverpool A	Liverpool B	Combined	*p*
	(*n* = 232)	(*n* = 66)	(*n* = 148)	(*n* = 446)	
p16/HPV status	–	–	–	–	<0.0001
p16−	137 (59%)	31 (47%)	44 (30%)	212 (48%)	–
p16+/HPV+	85 (37%)	31 (47%)	98 (66%)	214 (48%)	–
p16+/HPV−	10 (4%)	4 (6%)	6 (4%)	20 (4%)	–
Age (years)	–	–	–	–	0.4617
0–59	118 (51%)	39 (59%)	86 (58%)	243 (54%)	–
60+	114 (49%)	27 (41%)	62 (42%)	203 (46%)	–
Sex	–	–	–	–	0.8385
Male	174 (75%)	51 (77%)	117 (79%)	342 (77%)	–
Female	58 (25%)	15 (23%)	31 (21%)	104 (23%)	–
Tumour site of origin	–	–	–	–	0.0009
Tonsil	98 (42%)	40 (61%)	68 (46%)	206 (46%)	–
Base of tongue	60 (26%)	9 (14%)	56 (38%)	125 (28%)	–
Oropharynx (unless otherwise specified)	74 (32%)	17 (26%)	24 (16%)	115 (26%)	–
Smoking status	–	–	–	–	<0.0001
Never	26 (11%)	11 (17%)	37 (25%)	74 (17%)	–
Previous	57 (25%)	33 (50%)	62 (42%)	152 (34%)	–
Current	94 (41%)	21 (32%)	47 (32%)	162 (36%)	–
Missing	55 (24%)	1 (2%)	2 (1%)	58 (13%)	–
Alcohol history	–	–	–	–	<0.0001
No	36 (16%)	13 (20%)	107 (72%)	156 (35%)	–
Yes	110 (47%)	51 (77%)	39 (26%)	200 (45%)	–
Missing	86 (37%)	2 (3%)	2 (1%)	90 (20%)	–
Surgery with curative intent	–	–	–	–	0.0004
Yes	139 (60%)	60 (91%)	96 (65%)	295 (66%)	–
No	68 (29%)	6 (9%)	43 (29%)	117 (26%)	–
No treatment/palliative	25 (11%)	0 (0%)	9 (6%)	34 (8%)	–
TNM7	–	–	–	–	0.7022
I	21 (9%)	4 (6%)	9 (6%)	34 (8%)	–
II	33 (14%)	6 (9%)	12 (8%)	51 (11%)	–
III	39 (17%)	11 (17%)	23 (16%)	73 (16%)	–
IV	139 (60%)	45 (68%)	104 (70%)	288 (65%)	–
TNM8	–	–	–	–	0.0898
I	37 (16%)	10 (15%)	20 (14%)	67 (15%)	–
II	85 (37%)	26 (39%)	76 (51%)	187 (42%)	–
III	43 (19%)	9 (14%)	30 (20%)	82 (18%)	–
IV	67 (29%)	21 (32%)	22 (15%)	110 (25%)	–

Data is presented as number of patients (%). Differences in patient characteristics between the single study cohorts and the combined cohort were compared using Fisher’s exact test for categorical variables

### p16+/HPV− patients share similar baseline characteristics to p16+/HPV+ patients but are more likely to be older and not receive curative surgery

Approximately half (52%) of patients were p16 IHC positive. Of the 232 p16+ patients assessed, 20 (9%) did not test positive for HPV RNA. These 20 patients were also negative for HPV DNA-ISH confirming there was no bias due to differences in the type of RNA ISH probe used between the two study sites. Incidence of p16+/HPV+ and p16+/HPV− OPSCC was more common in males, previous smokers and those presenting with advanced stage according to TNM7 and stage II when adjusted using TNM8 (*p* < 0.05), Table 2. Patients with p16+/HPV+ OPSCC were more likely to be under 60 years of age and to have received surgery with curative intent than p16− or p16+/HPV− patients. p16+/HPV− patients share similar baseline characteristics to p16+/HPV+ patients, including smoking habits and tumour site of origin, but are more likely to be older and not receive curative surgery for treatment.

**Table 2:** Baseline characteristics of study patients in the combined cohort, according to p16/HPV status

	p16/HPV status			
	p16−	p16+/HPV+	p16+/HPV−	*p*
	(*n* = 212)	(*n* = 214)	(*n* = 20)	
Age (years)	–	–	–	<0.0001
0–59	94 (44%)	145 (68%)	4 (20%)	–
60+	118 (56%)	69 (32%)	16 (80%)	–
Sex	–	–	–	0.0478
Male	152 (72%)	175 (82%)	15 (75%)	–
Female	60 (28%)	39 (18%)	5 (25%)	–
Tumour site of origin	–	–	–	<0.0001
Tonsil	56 (26%)	141 (66%)	9 (45%)	–
Base of tongue	66 (31%)	53 (25%)	6 (30%)	–
Oropharynx (unless otherwise specified)	90 (42%)	20 (9%)	5 (25%)	–
Smoker status	–	–	–	<0.0001
Never	10 (5%)	62 (29%)	2 (10%)	–
Previous	50 (24%)	94 (44%)	8 (40%)	–
Current	118 (56%)	40 (19%)	4 (20%)	–
Missing	34 (16%)	18 (8%)	6 (30%)	–
Alcohol history	–	–	–	0.0002
No	55 (26%)	96 (45%)	5 (25%)	–
Yes	108 (51%)	85 (40%)	7 (35%)	–
Missing	49 (23%)	33 (15%)	8 (40%)	–
Surgery with curative intent	–	–	–	<0.0001
Yes	121 (57%)	162 (76%)	12 (60%)	–
No	65 (31%)	47 (22%)	5 (25%)	–
No treatment/palliative	26 (12%)	5 (2%)	3 (15%)	–
TNM7	–	–	–	<0.0001
I	31 (15%)	2 (1%)	1 (5%)	–
II	37 (17%)	12 (6%)	2 (10%)	–
III	36 (17%)	35 (16%)	2 (10%)	–
IV	108 (51%)	165 (77%)	15 (75%)	–
TNM8	–	–	–	<0.0001
I	31 (15%)	32 (15%)	4 (20%)	–
II	37 (17%)	140 (65%)	10 (50%)	–
III	36 (17%)	40 (19%)	6 (30%)	–
IV	108 (51%)	2 (1%)	0 (0%)	–

Data is presented as number of patients (%). Differences in patient characteristics according to p16/HPV status were compared using Fisher’s exact test for categorical variables

### Mortality of p16+/HPV− patients is similar to p16− patients but TNM8 downstages 95% of p16+/HPV− patients to early stage disease

**Fig. 1:**
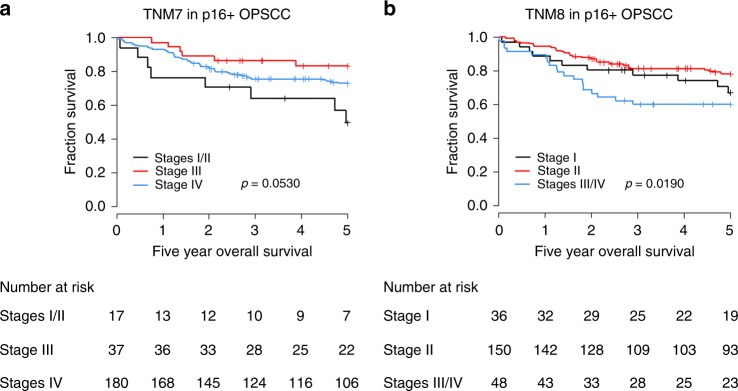
Kaplan–Meier estimates of five-year overall survival by (**a**) TNM7 and (**b**) TNM8 for p16+ OPSCC Global differences in survival curves were compared through use of the log-rank test

**Fig. 2:**
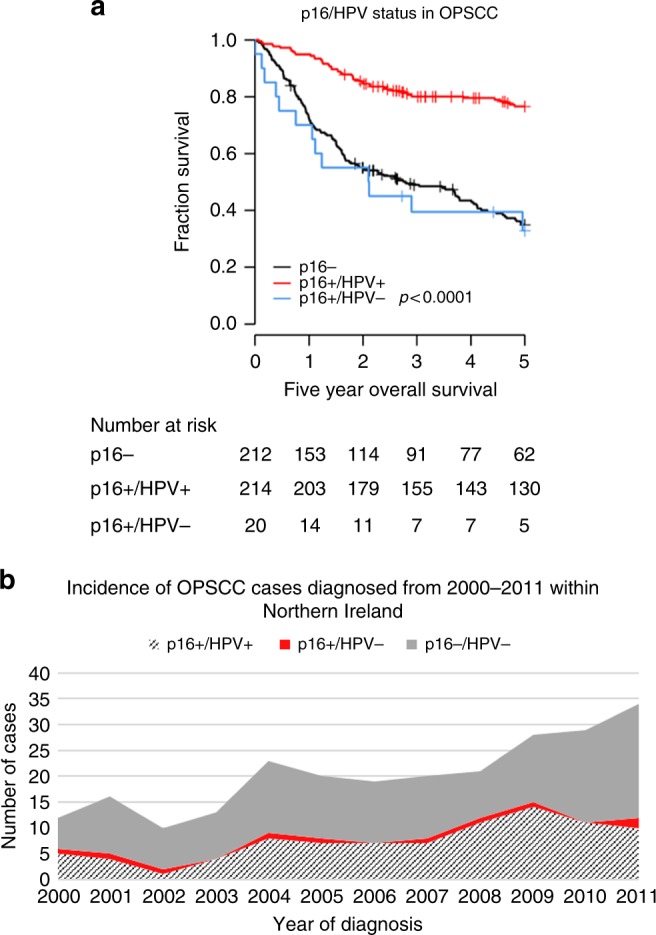
**a** Kaplan–Meier estimates of five-year overall survival by p16/HPV status in the combined cohort and (**b**) Time trend analysis of p16 and HPV status in OPSCC patients diagnosed in Northern Ireland during the study period Global differences in survival curves were compared through use of the log-rank test

Use of TNM8 staging improved upon TNM7 in all p16+ patients within the combined cohort. TNM8 but not TNM7 showed significant differences in overall survival by stage; with only 60% (95% CI: 47.96–75.93) of p16+ patients staged III/IV under TNM8 alive after five years compared to 67% (95% CI: 52.63–85.38) and 78% (95% CI: 71.71–85.39) of stage I and II patients, respectively, Fig. 1a, b. Restaging under TNM8 as p16+OPSCC reduced the patient’s stage in 95% of patients with p16+/HPV− OPSCC. Patient mortality of p16+/HPV− patients was similar to p16− patients; with only 33% (95% CI: 17.00–63.30) and 35% (95% CI: 28.81–42.32) of patients alive after five years, respectively, compared to 77% (95% CI: 70.96–82.66) of patients with p16+/HPV+OPSCC, Fig. 2a. HPV status was found to be an independent survival predictor within p16+ OPSCCs, with HPV− patients having a two-fold increased risk of death (HR .66 [95% CI: 1.37–5.15]) compared to HPV+patients, Table 3. Prevalence of p16+/HPV+and p16+/HPV− OPSCC within the population based Northern Ireland cohort was found to be relatively consistent over the study period with an average 40% of OPSCC being diagnosed as p16+ each year; 86% of these were HPV+ and 14% HPV−, Fig. 2b. To summarise, mortality of p16+/HPV− patients was similar to p16− patients but TNM8 downstages 95% of p16+/HPV− patients to early stage disease.

**Table 3:** Multivariable analysis of study patients in the combined cohort

	Univariate	*p*	Multivariable	*p*
	Hazard ratio (95% CI)		Hazard ratio (95% CI)	
p16/HPV status				
p16+/HPV+	Reference	–	Reference	–
p16+/HPV−	4.40 (2.39–8.13)	<0.0001	2.66 (1.37–5.15)	0.0037
p16−	3.86 (2.77–5.38)	<0.0001	2.03 (1.27–3.26)	0.0033
Age (years)				
0–59	Reference	–	Reference	–
60	2.00 (1.51–2.67)	<0.0001	1.48 (1.09–2.01)	0.0126
Sex				
Male	Reference	–	Reference	–
Female	0.83 (0.59–1.18)	0.3030	0.73 (0.51–1.05)	0.0883
Tumour site of origin				
Tonsil	Reference	–	Reference	–
Base of tongue	1.94 (1.39–2.72)	0.0001	1.08 (0.75–1.55)	0.6903
Oropharynx (unless otherwise specified)	1.76 (1.24–2.50)	0.0017	0.99 (0.66–1.47)	0.9568
Smoker status				
Never	Reference	–	Reference	–
Previous	1.44 (0.83–2.51)	0.1970	1.04 (0.59–1.83)	0.9045
Current	3.27 (1.95–5.49)	<0.0001	1.41 (0.80–2.49)	0.2358
Missing	3.79 (2.12–6.77)	<0.0001	1.26 (0.60–2.64)	0.5359
Alcohol history				
No	Reference	–	Reference	–
Yes	1.89 (0.85–1.66)	0.3103	0.96 (0.67–1.38)	0.8357
Missing	1.96 (1.34–2.86)	0.0005	1.56 (0.93–2.61)	0.0938
Surgery with curative intent				
Yes	Reference	–	Reference	–
No	2.10 (1.53–2.87)	<0.0001	1.71 (1.22–2.39)	0.0017
No treatment/palliative	7.72 (5.08–11.73)	<0.0001	3.74 (2.35–5.95)	<0.0001
TNM8				
I	Reference	–	Reference	–
II	0.71 (0.44–1.15)	0.1680	0.97 (0.59–1.62)	0.9159
III	1.31 (0.79–2.18)	0.3020	1.23 (0.73–2.07)	0.4394
IV	3.26 (2.07–5.11)	<0.0001	1.93 (1.18–3.17)	0.0093

Data are hazard ratios (95% CI) and corresponding *p* values. Models were mutually adjusted for each variable included in the table using pairwise comparison for the reference category in each covariate

## Discussion

Inclusion of solitary p16 IHC testing with TNM8 improves capability to appropriately stage OPSCC for prognostic stratification but it critically fails to recognise patients who lack HPV aetiology and carry significantly inferior clinical outcome. Interrogation of HPV status using RNA-ISH within p16+ OPSCC identified a subpopulation of p16+ patients who had a two-fold increased risk of death following adjustment for potential confounders. These p16+/HPV− patients were more similar to patients with demonstrably HPV− disease and would stand to benefit from current standards of treatment intensity. Importantly, when the newly proposed TNM8 staging guidelines were applied we found that 95% of these p16+/HPV− patients would be down-staged in circumstances where p16 IHC alone was used with no additional confirmatory ISH test. Due to selection bias in the cohorts utilised only 9% of p16+ OPSCC patients were HPV− in the combined cohort. However, when considered in the context of a population-based cohort, this subpopulation was equivalent to 11% of p16+ OPSCC patients diagnosed in Northern Ireland over a ten-year period. These findings carry significant implications for evolving treatment algorithms if HPV testing guidelines for patient management follow precedent set by TNM8 for oropharyngeal cancers; failure to confirm true HPV status could result in inappropriate de-escalation or sub-optimal therapy for these p16+/HPV− patients.

TNM7 staging guidelines did not take into account prognostic differences between HPV positive and negative cancers arising in the oropharynx. Indeed, the use of TNM7 was found to be insufficient for staging patients with p16+OPSCC in our study, a finding substantiated within the literature.^20^ TNM8 guidelines were developed to take account of variations in prognosis within OPSCC based on HPV status. Use of p16 IHC alone, as per TNM8 staging guidelines, demonstrated improved prognosis in our study but with overlap of stage I and II cancers as reported in some but not all studies.^21–25^ As part of an ‘organ preservation’ strategy the primary treatment modality for the majority of patients with OPSCC has been radiotherapy with and without concurrent chemotherapy and as such TNM8 staging criteria has been evaluated primarily in these patients.^13,20^ Notably our study and other studies demonstrating overlap of stages I and II included cohorts where the majority of patients received surgery and where use of pathologic TNM8 staging was recommended instead of clinical.^23–26^ Use of clinical TNM8 staging in retrospective cohorts of surgically managed patients may result in bias producing the overlap of stage I and II patients.

Following the precedent set by the UICC, the College of American Pathologists have updated their clinical recommendations for HPV testing and now advocate the use of p16 IHC as a standalone test, citing insufficient evidence of clinical need for a second line test in oropharyngeal primaries.^6^ p16 IHC was chosen for use in TNM8 as it is more cost effective and accessible to use without specialist molecular tests however its use in isolation does not meet the UK Royal College of Pathologists’ recommended guidelines, under which our study is modelled.^7,12^ At present, HPV assessment diagnostically for OPSCC is based on FFPE samples. As the samples available were not fresh frozen, use of RT-PCR for HPV detection could not be reliably used to confirm HPV status in p16+/HPV− samples. The HPV RNAscope assay has been demonstrated to be both optimally sensitive and specific for HPV detection in FFPE tissue but does not have fully regulatory approval for clinical use.^16,9,27^ We thereby eliminated potential bias in HPV RNA-ISH detection between Northern Ireland and Liverpool samples by testing all patient FFPE material that were not positive for p16 IHC and HPV RNA-ISH with clinically approved HPV DNA-ISH testing for FFPE samples. This established if any HPV DNA from 12 high-risk genotypes was present in the tumour tissue. No patient who was p16+/HPV RNA− was found positive for HPV DNA thereby eliminating any potential bias in our study because of the probes utilised.

We found the strategy of using p16 IHC followed by HPV RNA-ISH has allowed identification of all clinically relevant incidences of transcriptionally active HPV+ tumours within our cohort and is supported by recent meta-analyses which have demonstrated that a two tiered test is essential for a reliable diagnosis.^28^ The College for American Pathologists finds use of p16 IHC in patients with primary OPSCC to be sufficient for molecular stratification of HPV in the head and neck.^6^ Interestingly, the majority of p16+/HPV+ and p16+/HPV− tumours in our study originated in either base of tongue or tonsil, supporting a recent meta-analysis, but we found the oropharyngeal sub-site did not confer survival advantage when adjusted for covariates.^29^ These data demonstrate that p16 IHC appropriately classified 91% of HPV+ patients, a finding substantiated by recent meta-analyses within the literature for head and neck cancers.^3^ Critically, we find that the resulting 9% of patients who are p16+/HPV− are of increasing clinical importance and concern as these patients have significantly reduced survival compared to p16+/HPV+ patients and would not benefit from TNM8 staging guidelines; as 95% of patients with p16+/HPV− tumours were found to be down-staged when assessed using clinical TNM8 as HPV+ OPSCC.

Based on the results of the current study we find that ongoing clinical trials for de-intensification of treatment in OPSCC patients, which utilise p16 IHC only to stratify patients, carry potential for p16+/HPV− patients to receive sub-optimal treatment as treatment algorithms evolve.^30,31^ These findings are supported in a recent study of OPSCC and stage, which utilised HPV DNA-ISH and p16 IHC.^32,33^ In recognising the importance of a second line test to prevent sub-optimal treatment of transcriptionally inactive HPV tumours we find confirmation of HPV status would result in more appropriate prognostication of p16+/HPV− patients and a better staging tool overall for OPSCC.

To conclude, our study provides compelling evidence that lack of a standardised two-tiered HPV testing process, when using p16 IHC will negatively affect the clinical management of p16+/HPV− patients as treatment algorithms evolve. When p16 IHC is utilised as a first line-screening test, subsequent confirmation of HPV status based on definitive HPV detection using ISH must follow.

## Data Availability

Data is held within the Northern Ireland Biobank and the University of Liverpool Department of Molecular and Clinical Cancer Medicine and is available on application.
